# Wide-Angle Emission in Cylindrical Moiré Lattices Enabled by Rolling Origami

**DOI:** 10.1021/acs.nanolett.6c00692

**Published:** 2026-04-13

**Authors:** Min Tang, Fanzhou Lv, Haiyun Dong, Jiawei Wang, Chaoyuan Jin, Tun Cao, Ching Hua Lee, Ronny Thomale, Sebastian Klembt, Yana Vaynzof, Libo Ma

**Affiliations:** † School of Optoelectronics Engineering and Instrumentation Science, 12399 Dalian University of Technology, Dalian 116024, China; ‡ 28394 Leibniz Institute for Solid State and Materials Research Dresden, 01069 Dresden, Germany; § Key Laboratory of Photochemistry, 53030 Institute of Chemistry, Chinese Academy of Sciences, Beijing 100190, China; ∥ School of Chemical Sciences, University of Chinese Academy of Sciences, Beijing 100049, China; ⊥ School of Integrated Circuits, 529484 Harbin Institute of Technology (Shenzhen), Shenzhen 518055, China; # College of Information Science and Electronic Engineering, 12377 Zhejiang University, Hangzhou 310027, China; ∇ Department of Physics, 37580 National University of Singapore, 117551 Singapore, Republic of Singapore; ○ Institut für Theoretische Physik und Astrophysik, 9190 Universität Würzburg, 97074 Würzburg, Germany; ● Technische Physik, Wilhelm-Conrad-Röntgen-Research Center for Complex Material Systems, 9190 Universität Würzburg, D-97074 Würzburg, Germany; ▲ Chair for Emerging Electronic Technologies, 9169 Technical University of Dresden, 01187 Dresden, Germany

**Keywords:** moiré lattice, self-rolling, cylindrical architecture, wide-angle emission

## Abstract

Twisted photonic lattices that form moiré superlattices have attracted significant attention owing to their unique properties, in which the localized optical modes can serve as efficient light sources. However, in conventional moiré lattices, the emission direction of confined modes is typically fixed, and achieving a broad range of emission angles remains a significant challenge. Here, we design and fabricate single-layer moiré photonic lattices into cylindrical geometries using a nanomembrane origami technique. This approach enables wide-angle localized-mode emission while maintaining stable single-mode operation and excellent spectral uniformity. The moiré supercells support localized flat-band modes under various effective twist angles, resulting in the observation of periodic localized-mode emission over a wide range of azimuthal angles. Our research provides an approach for developing moiré light sources on curved surfaces, offering significant potential in applications that demand spatial light control, including three-dimensional imaging, light detection and ranging, and topological state manipulation.

## Graphical abstract

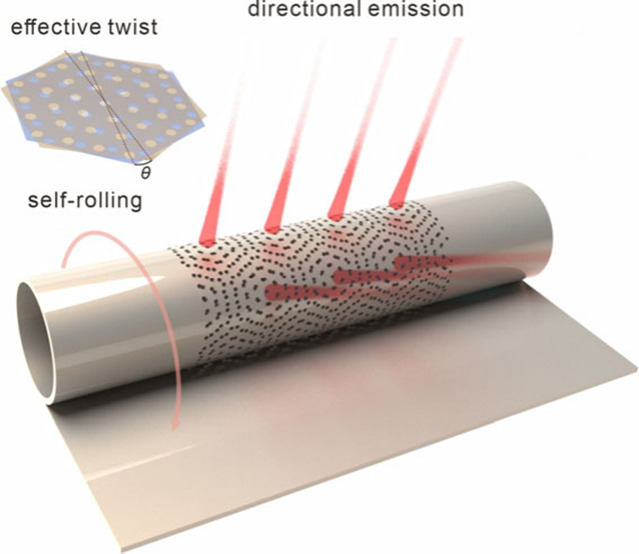

Twisted van der Waals heterostructures, formed by stacking two-dimensional atomic layers at a relative angle, have recently become an active research field due to their unique structural characteristics and the emergence of fascinating phenomena in these structures. The initial focus of related research was on graphene-based systems,^1^ where twisting two layers to a specific angle leads to the occurrence of flat bands.^2,3^ These flat bands give rise to strongly correlated electronic phenomena, such as Mott insulating states and unconventional superconductivity.^4–6^ This powerful “magic-angle” concept has been successfully transferred to optics by twisting two photonic crystal slabs, which form a moiré superlattice.^7–10^ The resulting moiré superlattice creates a periodic potential for light, thereby introducing a new degree of freedom for manipulating electromagnetic fields. Analogous to their electronic counterparts, these twisted photonic structures provide a robust theoretical framework for investigating phenomena like the formation of photonic flat bands,^11–13^ localization of photonic states,^14–17^ polarization control,^18,19^ frequency tuning of the band structure,^20^ and the engineering of bound states in the continuum.^21,22^

As a key factor in photonic moiré superlattices, optical coupling between photonic crystal modes is typically achieved through the fine-tuning of the gap between photonic crystal layers^7,23^ or by employing spatial light interference.^24^ A more convenient approach proposes a single layer designed as the effective stacking of two photonic crystal layers.^8^ This method is favored for its simplicity and compatibility with standard planar semiconductor processes. In this type of moiré superlattice, the unique properties yielded from moiré flat bands, such as high quality (*Q*) factors and small mode volume, have led to significant applications, including the demonstration of moiré lasers^8,25^ and novel platforms for cavity quantum electrodynamics.^26,27^ Although moiré photonic crystals have been extensively investigated in both fundamental research and experimental demonstrations, their spatial emission directionality remains limited due to the lack of effective strategies for tuning the fixed architecture. Although a pioneering method can tune emission direction by modulating the phase in individual unit cells,^28^ achieving a wide range of emission angles with a simple modulation scheme remains a significant challenge.

Nanomembrane origami offers a convenient approach for creating 3D microstructures from 2D nanomembranes, providing exceptional flexibility in designing functional micro- and nano- photonic devices.^29^ Among the various microstructures created using the origami technique, self-rolled-up microtubular structures represent a versatile platform that can support efficient 3D light confinement and resonances, attracting broad scientific attention.^30^ This type of microtubular photonic structure demonstrates significant potential in both fundamental physics and applications across various fields, including many-body coupling systems,^31–33^ geometric phase generation,^34^ in-plane to out-of-plane mode coupling,^35^ optoplasmonics,^36^ and laser technologies.^37^ As a 3D architectural system, the microtubular structure not only offers an excellent foundation for manipulating optical fields and realizing spatially controllable light emission at the microscale, but also provides a promising route toward the development of multifunctional integrated photonic devices.

In the present work, single-layer moiré photonic lattices are designed and fabricated into a cylindrical structure using a nanomembrane origami method. This approach enables the realization of a wide-angle range of localized-mode emission due to the formation of photonic flat bands in the cylindrically structured moiré lattices. The moiré supercells can support localized flat band modes under various effective twist angles. Periodic localized-mode emission has been observed that directly corresponds to the underlying lattice periodicity. The characterization of the two-dimensional emission intensity distribution shows excellent agreement with the simulation results. A series of cylindrical moiré photonic lattices with varying effective twist angles was fabricated to investigate the wide-angle range of localized-mode emissions. The spectral consistency and intensity uniformity of the localized modes were experimentally demonstrated across different azimuthal directions in cylindrical moiré superlattices. Furthermore, the proposed system’s design is compatible with batch fabrication, allowing the facet orientation of each unit cell to be flexibly designed and tuned on a chip. These cylindrical moiré superlattices thus hold great promise for enabling full angular coverage of light emission, thereby facilitating advancements in on-chip photonic applications.

As shown in Figure 1(a), the moiré pattern on a cylindrical structure is defined by two sets of triangular lattices of nanoholes. The translational periodicity of the resultant moiré pattern is determined by the angular misalignment (i.e., the effective twist angle θ) between the two triangular lattices. The cylindrical moiré superlattice is fabricated by the self-rolling of a 2D moiré superlattice layer, which is triggered by releasing a prestrained nanomembrane. As shown in Figure 1(b), the sample fabrication process consists of two primary steps: the initial patterning of nanomembranes on a planar wafer and the subsequent dry-release of the strained nanomembrane in plasma. In brief, a sacrificial layer of silicon (570 nm thick), a strained nanomembrane of SiN_*x*_ (140 nm thick), and a protective layer of Al_2_O_3_ (2 nm thick) were sequentially deposited on a SiO_2_/Si substrate. The boundaries of the rolling up of nanomembranes and the moiré lattice regions were defined using maskless lithography alignment and electron beam lithography, respectively. Subsequently, additional Al_2_O_3_ deposition and etching were performed to create an etching window in the silicon sacrificial layer. After etching away the sacrificial layer, the moiré lattice rolls up into a cylindrical structure by releasing the strain in the SiN_*x*_ nanomembrane. A detailed description of the fabrication process is provided in the Supporting Information. Figure 2(c) presents optical microscope images of the rolled-up moiré superlattices. The gray region at the center of the microtube corresponds to the area of the photonic moiré lattice. Figure 2(d) shows a moiré superlattice array with a distinct periodic pattern on the rolled-up microstructure. This self-rolling method enables the transformation of a 2D planar lattice into a curved lattice in 3D space.

**Figure 1:**
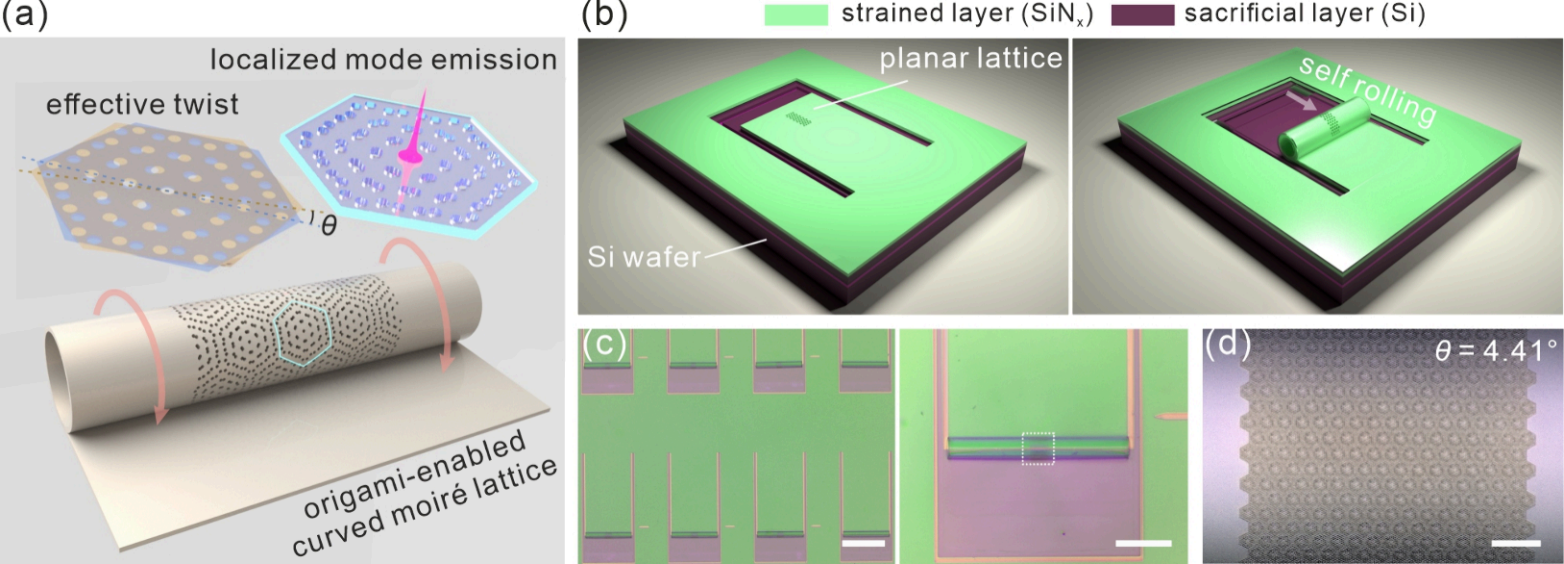
(a) Schematic diagram of moiré superlattice self-rolling into a cylindrical structure. The photonic moiré pattern is formed by effectively stacking two honeycomb photonic crystals at a twist angle θ. (b) Schematic diagram showing the two primary fabrication steps of rolling up a moiré superlattice: the initial patterning of nanomembranes on a planar wafer (left panel) and the subsequent dry release of the strained nanomembrane in plasma (right panel). (c) Bright-field microscopy images of the rolled-up cylindrical structures. The region within the black frame denotes the area of the moiré lattice. (d) Optical image of a cylindrical moiré superlattice with an effective twist angle of 4.41°. The scale bars are 300, 100, and 10 μm, respectively.

**Figure 2:**
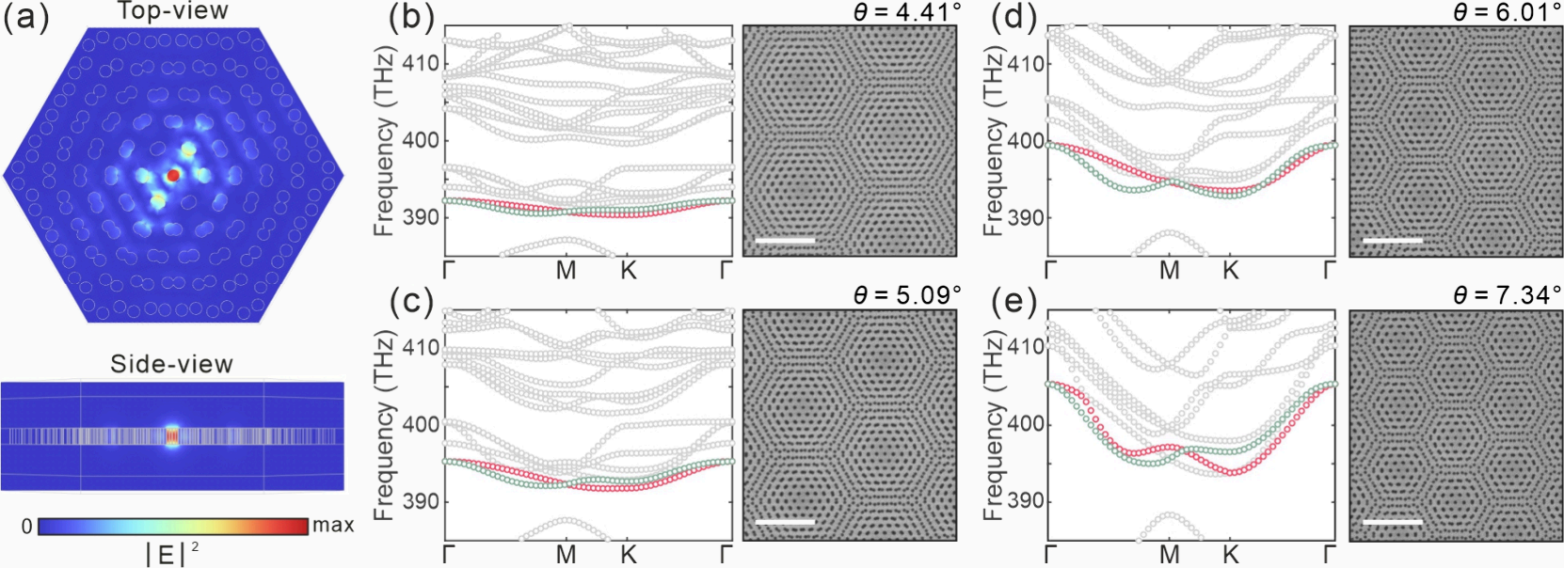
(a) 3D distribution of the localized flat band mode |*E*|^2^ in a single-layer moiré superlattice at an effective twist angle of 6.01°, with cross-sectional views in the top-view and side-view planes. SEM images of the moiré superlattice at the twisted angle of (b) 4.41°, (c) 5.09°, (d) 6.01°, and (e) 7.34° and the corresponding band structures. Scale bar: 2 μm.

To form a periodic Moiré superlattice, the twist angle θ is given by^38,39^

$$\theta =j\;\ln\left(\frac{2n_{1}+n_{2}+{jn}_{2}\sqrt{3}}{2m_{1}+m_{2}+{jm}_{2}\sqrt{3}}\right)$$ 1

In this study, a series of twist angles of 4.41°, 5.09°, 6.01°, and 7.34° are considered, corresponding to *n*_1_ = *m*_2_ = 8, 7, 6, 5 and *n*_2_ = *m*_1_ = 7, 6, 5, 4, respectively. An inverse relationship exists between the twist angle and the unit cell size, wherein larger angles yield smaller periodic structures. Differences in the unit cell size cause the localized optical modes to exhibit varying sensitivities to boundary conditions. Another critical parameter alongside the twist angle is the interlayer coupling strength. In the case of a merged single-layer moiré superlattice, this coupling strength reaches its maximum. In the context of flat band formation, interlayer coupling causes a repulsion of degenerate Bloch modes, pushing one set of the modes toward the Dirac cone and consequently forming a flat band.^8^ In the single-layer moiré superlattice, key parameters concern only the twist angle and the refractive index. In the previously reported moiré lattices,^8,25^ the refractive indices are greater than 2.5. In this work, the SiN_*x*_ membrane possesses a refractive index of around 2.0. The refractive index of the photonic crystal directly correlates with the intralayer coupling strength, with a lower refractive index resulting in a more pronounced intralayer coupling. Consequently, the relative interlayer coupling weakens. For the other key parameter, a relatively small twist angle would introduce more effective coupling sites within a single unit cell. Thus, a smaller twist angle corresponds to a flatter energy band, while a larger twist angle leads to a flat band with a broader frequency range for the localized modes. It is noteworthy that localized modes can be consistently formed with a similar distribution when varying the twist angle.

COMSOL Multiphysics is utilized to simulate mode profiles and band diagrams of the moiré superlattices. The moiré microcavity with refractive index distribution *n*(*x*,*y*) can be described by the scalar wave equation

$$-\nabla^{2}\varphi =n^{2}\left(\frac{\omega^{2}}{c^{2}}\right)\varphi$$ 2

where ω is the angular frequency, *c* is the light speed in vacuum, and φ represents the field distribution. Floquet boundary conditions are applied at the edges of the moiré superlattice. The original circular air hole possesses a radius of 55 nm and a lattice constant of 280 nm. The twist angles are set to 4.41°, 5.09°, 6.01°, and 7.34°, respectively. Three-dimensional simulations were performed to investigate the localized modes supported by the moiré superlattice. As shown in Figure 2(a), for a twist angle of 6.01°, the moiré superlattice exhibits strong optical confinement in both the in-plane and out-of-plane directions. Figures 2(b)–(e) present the SEM images of the moiré superlattices and the corresponding band structures at different twist angles. The flatness of the bands can be evaluated using the frequency Δ*f* = ω/2π. The obtained Δ*f* values are 1.86, 3.52, 6.61, and 11.56 THz for twist angles of 4.41°, 5.09°, 6.01°, and 7.34°, respectively, which are consistent with the expected correlation between the twist angle and flat-band formation.

For moiré superlattices with different twist angles, transverse electric mode is supported, with the predominant electric field component oriented in-plane. The mode field is primarily localized in the central air hole, exhibiting a rapid decay toward the lattice boundary. Due to the similar mode distributions, the corresponding spatial volumes or areas they occupy are nearly identical. In 2D simulations, the mode area *S*_*m*_ is used to characterize mode confinement ability, defined as

$$S_{m}=\frac{\int n^{2}{\left|\varphi \right|}^{2}\;dS}{\max\left(n^{2}{\left|\varphi \right|}^{2}\right)}$$ 3

The mode areas for the flat band modes are calculated to be 1.42, 1.30, 1.17, and 1.05 × (λ/*n*)^2^ for moiré superlattices with twist angles of 4.41°, 5.09°, 6.01°, and 7.34°, respectively, where λ is the wavelength of the mode, and *n* is the refractive index of SiN_*x*_. This indicates that the mode can be confined to a wavelength scale in the *xy*-plane, promoting stronger light-matter interactions.

The cylindrical moiré lattice with a twist angle of 4.41° was systematically measured due to its well-formed flat bands and localized modes. The measurements were performed using a laser confocal system. Figure 3(a) shows a typical spectrum of the localized mode, characterized by a single prominent peak with a full width at half-maximum (FWHM) of 0.28 nm, corresponding to a *Q* factor of 2.3 × 10^3^. The inset shows a two-dimensional mode intensity scan along the *x* and *y* directions with a mapping step of 300 nm, revealing that the mode exhibits excellent localization within an individual unit cell. To further characterize the periodicity of the mode, a line scan was conducted along the axial direction of the cylindrical structure. As illustrated in Figure 3(b), a periodic intensity distribution is revealed, with the observed period aligning precisely with the lattice periodicity.

**Figure 3:**
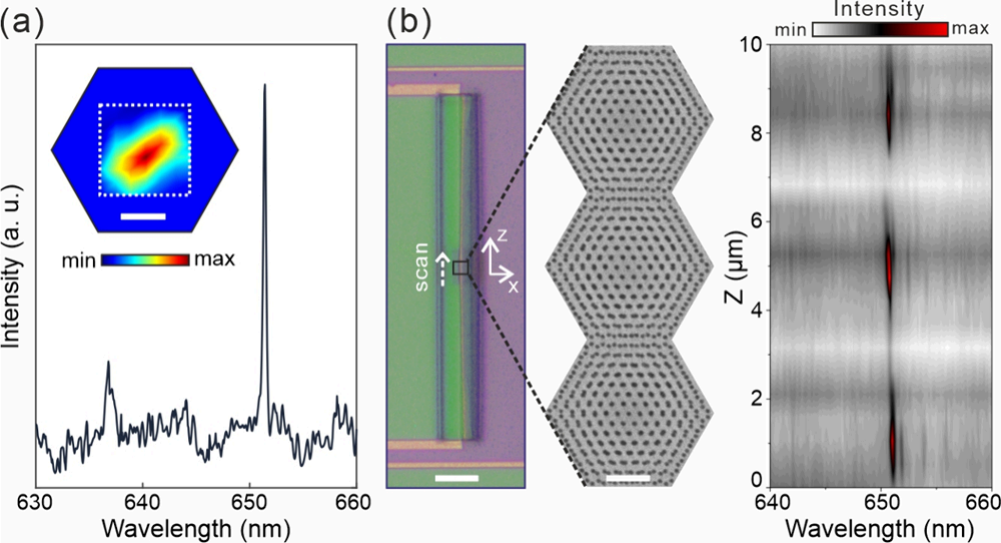
(a) Spectrum of the localized mode in a cylindrical moiré superlattice with a twist angle of 4.41°. Inset: experimental mapping of the mode within a single unit cell. The scale bar is 1 μm. (b) Spectra map of the localized mode showing its distribution across three periods of the superlattice. The scale bars from left to right are 40 μm and 1 μm, respectively.

Angle-dependent measurements were performed on the cylindrical moiré lattices to characterize their emission behavior, as illustrated in Figure 4(a). The tilt angle φ, defined as the angle between the incident laser beam and the surface normal of the substrate, varied from −30° to 30°. Four rolled-up moiré lattices with twist angles of 4.41°, 5.09°, 6.01°, and 7.34° were investigated under these conditions. Figure 4(b) presents the peak intensity map for moiré lattices with these twist angles as a function of tilt angle φ, where the peak intensities were normalized for each sample. The results reveal distinct localized emission modes characterized by sharp peaks in the wavelength range of 600–700 nm. The FWHM measurements indicate *Q* factors on the order of 2 × 10^3^. Detailed emission spectra for each lattice structure are shown in Figure 4(c) and 4(d), and the spectra for the structures with twist angles of 6.01° and 7.34° are provided in the Supporting Information (Figure S1­(a) and (b)). In addition to the central peak, several weak sidebands are observed, originating from weakly localized higher-order dipole modes. Moreover, as the twist angle decreases, the unit cell size increases, allowing more dipole modes to be supported, as shown in Figure 4(c). Overall, the spectra exhibit excellent uniformity in intensity, with a variation of less than 3 dB between the strongest and weakest peaks. The moiré lattice exhibits a reduced mode volume and exceptional single-mode characteristics owing to the low refractive index contrast and the curvature-induced suppression of nonlocal Floquet waves. Furthermore, the cylindrical moiré lattice shows great potential in achieving lasing by integrating with optical gain media, such as perovskites^37^ or other semiconductor nanostructures,^40,41^ thereby offering a promising pathway for the future development of spatial laser sources.

**Figure 4:**
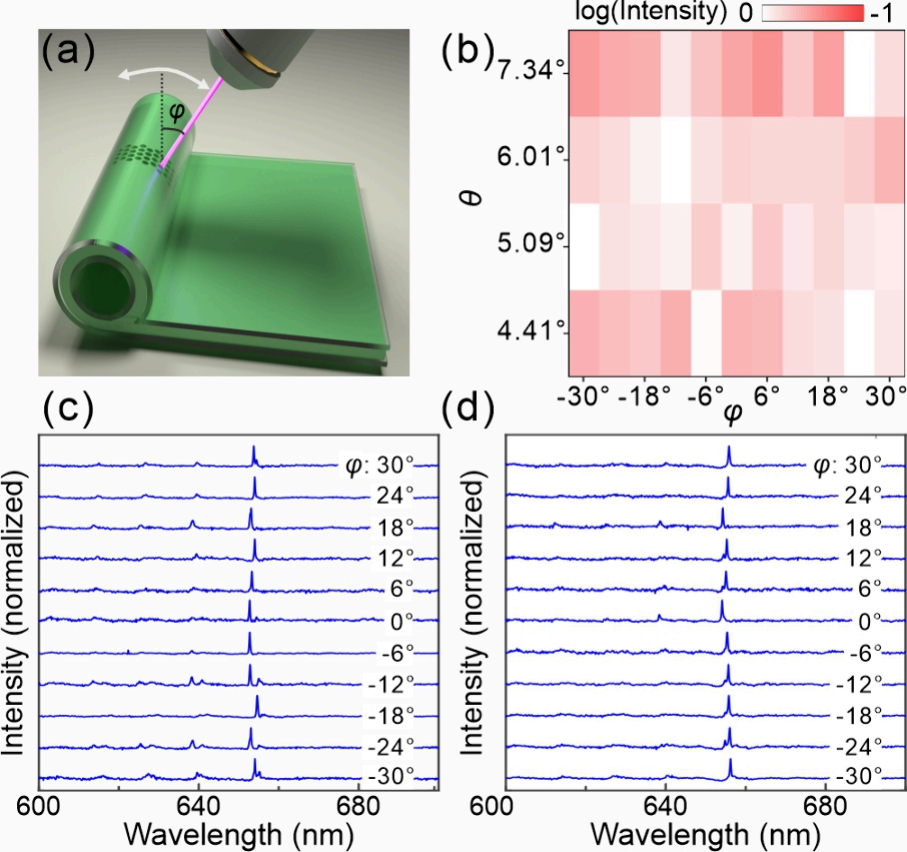
(a) Schematic illustration of angle-dependent measurement on a cylindrically structured moiré lattice. The tilt angle φ is defined as the angle between the incident laser beam and the surface normal of the substrate. (b) Peak intensity map of localized modes measured from cylindrical moiré lattices with different twist angles θ as a function of tilt angle φ. The measured emission spectra for cylindrical moiré lattices with twist angles of (c) 4.41° and (d) 5.09° are displayed with tilt angle φ varying from −30° to 30°. Weak sidebands are also observed in the short-wavelength range.

In summary, we have proposed and demonstrated a wide-angle range emission from localized modes in a single cylindrical moiré lattice. The system exhibits a robust localized mode with excellent spectral uniformity, as confirmed by band structure calculations, mode profile simulations, and spatial scan measurements. The observed consistency of emission characteristics across different orientations validates the wide-angle range of localized mode emissions. Beyond establishing a versatile platform for exploring moiré photonics on curved surfaces, this work provides a foundation for the development of next-generation photonic devices with controllable spatial emission, offering potential applications in integrated optical communication, imaging, and quantum information processing.

## Supplementary Material



## Notes

Details of sample preparation and emission spectra of moiré microtubes (PDF)

## References

[1] Novoselov K. S., Mishchenko A., Carvalho A., Castro Neto A. (2016). 2D Materials
and Van der Waals Heterostructures. Science.

[2] Cao Y., Fatemi V., Demir A., Fang S., Tomarken S. L., Luo J. Y., Sanchez-Yamagishi J.
D., Watanabe K., Taniguchi T., Kaxiras E., Ashoori R. C., Jarillo-Herrero P. (2018). Correlated
Insulator Behaviour at Half-Filling in Magic-Angle Graphene Superlattices. Nature.

[3] Cao Y., Fatemi V., Fang S., Watanabe K., Taniguchi T., Kaxiras E., Jarillo-Herrero P. (2018). Unconventional
Superconductivity
in Magic-Angle Graphene Superlattices. Nature.

[4] Chen G., Sharpe A. L., Gallagher P., Rosen I. T., Fox E. J., Jiang L., Lyu B., Li H., Watanabe K., Taniguchi T., Jung J., Shi Z., Goldhaber-Gordon D., Zhang Y., Wang F. (2019). Signatures of Tunable
Superconductivity
in a Trilayer Graphene Moiré Superlattice. Nature.

[5] Uri A., de la Barrera S. C., Randeria M. T., Rodan-Legrain D., Devakul T., Crowley P. J. D., Paul N., Watanabe K., Taniguchi T., Lifshitz R., Fu L., Ashoori R. C., Jarillo-Herrero P. (2023). Superconductivity and Strong Interactions in a Tunable
Moiré Quasicrystal. Nature.

[6] Tang H., Wang Y., Ni X., Watanabe K., Taniguchi T., Jarillo-Herrero P., Fan S., Mazur E., Yacoby A., Cao Y. (2024). On-Chip Multi-Degree-of-Freedom
Control of Two-Dimensional Materials. Nature.

[7] Tang H., Lou B., Du F., Zhang M., Ni X., Xu W., Jin R., Fan S., Mazur E. (2023). Experimental Probe of Twist Angle–Dependent
Band Structure of On-Chip Optical Bilayer Photonic Crystal. Sci. Adv..

[8] Mao X. R., Shao Z. K., Luan H. Y., Wang S. L., Ma R. M. (2021). Magic-Angle
Lasers in Nanostructured Moiré Superlattice. Nat. Nanotechnol..

[9] Wang P., Zheng Y., Chen X., Huang C., Kartashov Y. V., Torner L., Konotop V. V., Ye F. (2020). Localization and Delocalization
of Light in Photonic Moiré Lattices. Nature.

[10] Meng Y., Feng J., Han S., Xu Z., Mao W., Zhang T., Kim J. S., Roh I., Zhao Y., Kim D.-H., Yang Y., Lee J.-W., Yang L., Qiu C.-W., Bae S.-H. (2023). Photonic Van der Waals Integration
from 2D Materials to 3D Nanomembranes. Nat.
Rev. Mater..

[11] Dong K., Zhang T., Li J., Wang Q., Yang F., Rho Y., Wang D., Grigoropoulos C. P., Wu J., Yao J. (2021). Flatbands
in Magic-Angle Bilayer Photonic Crystals at Small Twists. Phys. Rev. Lett..

[12] Tang H., Du F., Carr S., DeVault C., Mello O., Mazur E. (2021). Modeling the
Optical Properties of Twisted Bilayer Photonic Crystals. Light. Sci. Appl..

[13] Tang H., Ni X., Du F., Srikrishna V., Mazur E. (2022). On-Chip Light Trapping
in Bilayer Moiré Photonic Crystal Slabs. Appl. Phys. Lett..

[14] Fu Q., Wang P., Huang C., Kartashov Y. V., Torner L., Konotop V. V., Ye F. (2020). Optical Soliton Formation
Controlled by Angle Twisting in Photonic Moiré Lattices. Nat. Photonics.

[15] Nguyen D. X., Letartre X., Drouard E., Viktorovitch P., Nguyen H. C., Nguyen H. S. (2022). Magic Configurations
in Moiré
Superlattice of Bilayer Photonic Crystals: Almost-Perfect Flatbands
and Unconventional Localization. Phys. Rev.
Res..

[16] Ni X., Liu Y., Lou B., Zhang M., Hu E. L., Fan S., Mazur E., Tang H. (2024). Three-Dimensional Reconfigurable
Optical Singularities in Bilayer Photonic Crystals. Phys. Rev. Lett..

[17] Saadi C., Nguyen H. S., Cueff S., Ferrier L., Letartre X., Callard S. (2024). How Many Supercells
Are Required for Unconventional
Light Confinement in Moiré Photonic Lattices?. Optica.

[18] Gromyko D., An S., Gorelik S., Xu J., Lim L. J., Lee H. Y. L., Tjiptoharsono F., Tan Z. K., Qiu C. W., Dong Z., Wu L. (2024). Unidirectional Chiral Emission via
Twisted Bilayer Metasurfaces. Nat. Commun..

[19] Lou B., Fan S. (2022). Tunable Frequency Filter
Based on Twisted Bilayer Photonic Crystal
Slabs. ACS Photonics.

[20] Lou B., Wang B., Rodríguez J. A., Cappelli M., Fan S. (2022). Tunable Guided
Resonance in Twisted Bilayer Photonic Crystal. Sci. Adv..

[21] Qin H., Chen S., Zhang W., Zhang H., Pan R., Li J., Shi L., Zi J., Zhang X. (2024). Optical Moiré
Bound States in the Continuum. Nat. Commun..

[22] Zhang T., Dong K., Li J., Meng F., Li J., Munagavalasa S., Grigoropoulos C. P., Wu J., Yao J. (2023). Twisted Moiré
Photonic Crystal Enabled Optical Vortex Generation Through Bound States
in the Continuum. Nat. Commun..

[23] Tang H., Lou B., Du F., Gao G., Zhang M., Ni X., Hu E., Yacoby A., Cao Y., Fan S. (2025). An Adaptive
Moiré Sensor for Spectro-Polarimetric Hyperimaging. Nat. Photonics.

[24] Wang P., Fu Q., Konotop V. V., Kartashov Y. V., Ye F. (2024). Observation of Localization
of Light in Linear Photonic Quasicrystals with Diverse Rotational
Symmetries. Nat. Photonics.

[25] Raun A., Tang H., Ni X., Mazur E., Hu E. L. (2023). GaN Magic-Angle
Laser in a Merged Moiré Photonic Crystal. ACS Photonics.

[26] Yan S., Li H., Yang J., Chen X., Liu H., Dai D., Zhu R., Ma Z., Shi S., Yang L. (2025). Cavity
Quantum Electrodynamics With Moiré Photonic Crystal Nanocavity. Nat. Commun..

[27] Wang Y.-T., Ye Q.-H., Yan J.-Y., Qiao Y., Liu Y.-X., Ye Y.-Z., Chen C., Cheng X.-T., Li C.-H., Zhang Z.-J. (2025). Moiré Cavity
Quantum Electrodynamics. Sci. Adv..

[28] Luan H. Y., Ouyang Y. H., Zhao Z. W., Mao W. Z., Ma R. M. (2023). Reconfigurable
Moiré Nanolaser Arrays with Phase Synchronization. Nature.

[29] Smela E., Inganäs O., Lundström I. (1995). Controlled Folding of Micrometer-Size
Structures. Science.

[30] Schmidt O. G., Eberl K. (2001). Thin Solid Films Roll
Up into Nanotubes. Nature.

[31] Wang X., Wang Z., Dong H., Saggau C. N., Tang H., Tang M., Liu L., Baunack S., Bai L., Liu J., Yin Y., Ma L., Schmidt O. G. (2022). Collective Coupling
of 3D Confined Optical Modes in Monolithic Twin Microtube Cavities
Formed by Nanomembrane Origami. Nano Lett..

[32] Saggau C. N., Valligatla S., Wang X., Dong H., Ma L., Schmidt O. G. (2022). Coaxial Micro-Ring Arrays Fabricated on Self-Assembled
Microtube Cavities for Resonant Light Modulation. Laser Photonics Rev..

[33] Wang J., Yin Y., Hao Q., Zhang Y., Ma L., Schmidt O. G. (2018). Strong
Coupling in a Photonic Molecule Formed by Trapping a Microsphere in
a Microtube Cavity. Adv. Opt. Mater..

[34] Ma L. B., Li S. L., Fomin V. M., Hentschel M., Gotte J. B., Yin Y., Jorgensen M. R., Schmidt O. G. (2016). Spin-Orbit Coupling of Light in Asymmetric Microcavities. Nat. Commun..

[35] Valligatla S., Wang J., Madani A., Naz E. S. G., Hao Q., Saggau C. N., Yin Y., Ma L., Schmidt O. G. (2020). Selective
Out-of-Plane Optical Coupling between Vertical and Planar Microrings
in a 3D Configuration. Adv. Opt. Mater..

[36] Yin Y., Li S., Böttner S., Yuan F., Giudicatti S., Saei Ghareh Naz E., Ma L., Schmidt O. G. (2016). Localized Surface
Plasmons Selectively Coupled to Resonant Light in Tubular Microcavities. Phys. Rev. Lett..

[37] Dong H., Saggau C. N., Zhu M., Liang J., Duan S., Wang X., Tang H., Yin Y., Wang X., Wang J., Zhang C., Zhao Y. S., Ma L., Schmidt O. G. (2021). Perovskite Origami for Programmable Microtube Lasing. Adv. Funct. Mater..

[38] Oudich M., Kong X., Zhang T., Qiu C., Jing Y. (2024). Engineered
Moiré Photonic and Phononic Superlattices. Nat. Mater..

[39] Martí-Sabaté M., Torrent D. (2021). Dipolar Localization
of Waves in Twisted Phononic Crystal
Plates. Phys. Rev. Appl..

[40] Dastjerdi M., Djavid M., Mi Z. (2015). An Electrically
Injected Rolled-Up
Semiconductor Tube Laser. Appl. Phys. Lett..

[41] Heo J., Bhowmick S., Bhattacharya P. (2012). Threshold
Characteristics of Quantum
Dot Rolled-Up Microtube Lasers. IEEE J. Quantum
Electron..

