# Supplementary material for: Wide-Angle Emission in Cylindrical Moiré Lattices Enabled by Rolling Origami
Source: Nano Lett. 2026 Apr 13;26(15):5245–50. doi: 10.1021/acs.nanolett.6c00692 (PMC13107502; doi:10.1021/acs.nanolett.6c00692)
Supplement: Supplementary file 1 [file nl6c00692_si_001.pdf]

Supporting Information for Wide-angle emission in cylindrical moiré lattices enabled by rolling origami

Min Tang,[1,2,*] Fanzhou Lv,[2] Haiyun Dong,[3,4] Jiawei Wang,[5] Chaoyuan Jin,[6] Tun Cao,[1] Ching Hua Lee,[7] Ronny Thomale,[8] Sebastian Klembt,[9] Yana Vaynzof,[2,10] Libo Ma[2,*]

[1]School of Optoelectronics Engineering and Instrumentation Science, Dalian University of Technology, Dalian, 116024, China

[2]Leibniz Institute for Solid State and Materials Research Dresden, 01069 Dresden, Germany

[3]Key Laboratory of Photochemistry, Institute of Chemistry, Chinese Academy of Sciences, Beijing 100190, China

[4]School of Chemical Sciences, University of Chinese Academy of Sciences, Beijing 100049, China

[5]School of Integrated Circuits, Harbin Institute of Technology (Shenzhen), Shenzhen 518055, China

[6]College of Information Science and Electronic Engineering, Zhejiang University, Hangzhou 310027, China

[7]Department of Physics, National University of Singapore, 117551 Singapore, Republic of Singapore

[8]Institut für Theoretische Physik und Astrophysik, Universität Würzburg, 97074 Würzburg, Germany

[9]Technische Physik, Wilhelm-Conrad-Röntgen-Research Center for Complex Material Systems, Universität Würzburg, D-97074 Würzburg, Germany

[10]Chair for Emerging Electronic Technologies, Technical University of Dresden, 01187 Dresden, Germany

Experimental Section

Substrates: 10 mm ×10 mm silicon on insulator (SOI) wafer with 2 µm buried thermal oxide (BOX) is used as the substrate.

Deposition: The deposition membrane structure consists of an Al_2O_3 stop layer/Si sacrificial layer/Al_2O_3 protecing layer/SiN_x strained layer from bottom to top. Both Al_2O_3 layers are 4 nm and were deposited by ALD (GEMStar XT, Arradiance LLC, Littleton, USA). Silicon layer functions as sacrifice layer and was deposited by plasma enhanced chemical vapor deposition (PECVD, SI 500 D, Sentech Instruments). The 140 nm SiN_x strained layer was deposited by the same PECVD tool while the strain is introduced by changing of the bias and ICP-power (ICP: 1200 W, 150V, 30 s,10 s/ICP:100 W 0 V, 230 s) as process gases silane and nitrogen were used (SiH_4 [5% in He]: 250 sccm, N_2: 80/8/80 sccm, Ar: 140 sccm, Time: 30s/10s/230s). After the MLA/EBL and following RIE process, another 2 nm Al_2O_3 layer was deposited for protecting SiN_x during sacrifice layer etching.

Patterning: AZ5214E (photoresist, Mircochemicals GmbH, Ulm, Germany) was spun onto the samples at 4500 rpm, which gives a thickness of 1.3 μm of the photoresist. Pattering was performed by a maskless aligner (MLA 100, Heidelberg Instruments Mikrotechnik GmbH, Heidelberg, Germany) at a dose of 90 mJ cm^{-2}. Then the sample was developed in AZ 726 MIF developer (Microchemicals) for 45s. SML 300 (EBL resist, EM resist LTD, Macclesfield, UK) was spun onto the samples (1700/6000 rpm, 60/3 s). The sample was exposed at 50 kV in an EBL system (Voyager, Raith, GmbH, Dortmund, Germany) utilizing a dose of 800 μC cm^{-2}. Then the sample was developed in AR 600-56 (Allresist) for 90 s (including 50 sultrasonic cleaning).

Etching: The moiré structures were etched in an ICP-RIE (Plasma Lab 100, Oxford Instruments plc, Abingdon, UK) with CHF_3: 20 sccm, CF_4:10 sccm, and O_2:6 sccm as reactive gases. ICP: 450 W, Bias:145 V, pressure: 0.01 mbar, 80 s. The membranes were structured by photolithography and ICP-RIE etching. For Al_2O_3 layer it was etched with BCl_3: 20 sccm, He:10 sccm, ICP: 400 W, Bias:80 V, pressure: 0.01 mbar, 60 s. For SiN_x layer it was etched with SF_6: 20 sccm, He:10 sccm, ICP: 400 W, Bias:15 V, pressure: 0.01 mbar, 180 s.

Release: Samples were released by etching Si sacrificial layer inside a xenon difluoride etching system (Xactix® e2; Orbotech Ltd., Yavne, Israel).

Sample Characterization: Scanning Electron Microscopy images were taken on a DSM982 GEMINI at 5 kV, (Carl Zeiss Microscopy GmbH, Jena, Germany), Optical characterization was performed with a confocal photoluminescence setup (LabRAM

HR Evolution, Horiba Ltd., Kyoto, Japan). A laser beam at an excitation wavelength of 457 nm was focused onto the microtube cavities surface through a long working distance objective lens.

Emission spectra of moiré microtubes with different titled angles

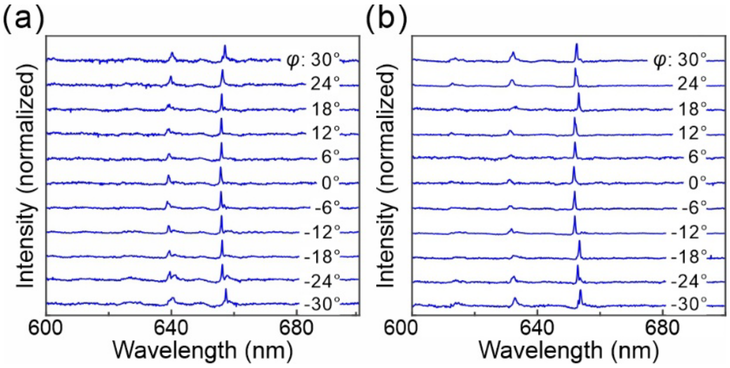

Figure S1. Detailed emission spectra of moiré microtubes with twist angles of (a) 6.01° and (b) 7.34°, measured with φ varying from -30° to 30°.
